# Recruiting patients with advanced malignant and non-malignant disease: lessons learned from a palliative care RCT

**DOI:** 10.1186/1745-6215-12-S1-A119

**Published:** 2011-12-13

**Authors:** Morag C Farquhar, Barbara Brafman-Kennedy, Irene J Higginson, Sara Booth

**Affiliations:** 1 Department of Public Health & Primary Care, University of Cambridge, Cambridge CB2 0SR, UK; 2 Palliative Care, Cambridge University Hospitals NHS Foundation Trust, Cambridge, CB2 0QQ, UK; 3 Department of Palliative Care, Policy & Rehabilitation, King’s College London, London, SE5 9PJ, UK

## Objectives

Recruiting patients to palliative care randomised controlled trials (RCTs) is particularly challenging. This paper describes and analyses the differing recruitment trajectories for patients with advanced malignant and non-malignant disease to a palliative care RCT, outlining activities undertaken to achieve targets. It will outline the lessons learned in order to inform design and conduct of future studies.

## Methods

Analysis of descriptive recruitment statistics (patient identification, response and completion rates) to a Phase II pilot pragmatic single-blind fast track RCT, and subsequent Phase III RCT, of a breathlessness intervention service for advanced disease. Phase II piloted with chronic obstructive pulmonary disease (COPD) patients only, whereas the Phase III RCT incorporated two sub-protocols: one for patients with malignant and one for non-malignant disease. Documentary analysis of: recruitment activity log, Trial Management and Advisory Group minutes and field notes.

## Results

Recruitment targets for patients with non-malignant disease were achieved. The pathway to recruitment was through referral to the service therefore referral rates impacted on recruitment alongside response rates. Recruitment of cancer patients was considerably slower despite concerted efforts to increase referrals by raising the service profile. Referrals only improved for the latter when a researcher attended clinics, supporting clinical staff in patient identification: recruitment tripled from 0.8 to 2.4 patients per month. Three possible reasons for the effectiveness of this are (1) dedicated time, (2) reciprocity & (3) established relationships. Predictably, response rates remained lower for patients with malignant disease than for those with non-malignant disease.

## Conclusions

Recruitment was partly referral-driven, therefore gate-keeping did not explain the differences. Clinical inter-professional relationships consolidated in Phases 0-II drove early referrals of non-malignant disease patients. Local palliative care services were available for patients with cancer. Consideration of the natural history and context of a service is therefore important when predicting recruitment. Pilot trials are informative, but should include qualitative elements and all disease groups. Placing researchers in relevant clinical settings is helpful.

